# Biochemical and spectroscopic characterization of purified Latex Clearing Protein (Lcp) from newly isolated rubber degrading *Rhodococcus rhodochrous* strain RPK1 reveals novel properties of Lcp

**DOI:** 10.1186/s12866-016-0703-x

**Published:** 2016-05-23

**Authors:** Sirimaporn Watcharakul, Wolf Röther, Jakob Birke, Kamontam Umsakul, Brian Hodgson, Dieter Jendrossek

**Affiliations:** Institute of Microbiology, University of Stuttgart, Allmandring 31, 70569 Stuttgart, Germany; Prince of Songkla University, Songkla, Thailand

**Keywords:** Latex clearing protein (Lcp), Rubber oxygenase, Dioxygenase, Rhodococcus, Biodegradation

## Abstract

**Background:**

Biodegradation of rubber (polyisoprene) is initiated by oxidative cleavage of the polyisoprene backbone and is performed either by an extracellular rubber oxygenase (RoxA) from Gram-negative rubber degrading bacteria or by a latex clearing protein (Lcp) secreted by Gram-positive rubber degrading bacteria. Only little is known on the biochemistry of polyisoprene cleavage by Lcp and on the types and functions of the involved cofactors.

**Results:**

A rubber-degrading bacterium was isolated from the effluent of a rubber-processing factory and was taxonomically identified as a *Rhodococcus rhodochrous* species. A gene of *R. rhodochrous* RPK1 that coded for a polyisoprene-cleaving latex clearing protein (*lcp*_*Rr*_) was identified, cloned, expressed in *Escherichia coli* and purified. Purified Lcp_Rr_ had a specific activity of 3.1 U/mg at 30 °C and degraded poly(1,4-*cis*-isoprene) to a mixture of oligoisoprene molecules with terminal keto and aldehyde groups. The pH optimum of Lcp_Rr_ was higher (pH 8) than for other rubber-cleaving enzymes (≈ pH 7). UVvis spectroscopic analysis of Lcp_Rr_ revealed a cytochrome-specific absorption spectrum with an additional feature at long wavelengths that has not been observed for any other rubber-cleaving enzyme. The presence of one *b*-type haem in Lcp_Rr_ as a co-factor was confirmed by (i) metal analysis, (ii) solvent extraction, (iii) bipyridyl assay and (iv) detection of haem-*b* specific *m/z* values via mass-spectrometry.

**Conclusions:**

Our data point to substantial differences in the active sites of Lcp proteins obtained from different rubber degrading bacteria.

**Electronic supplementary material:**

The online version of this article (doi:10.1186/s12866-016-0703-x) contains supplementary material, which is available to authorized users.

## Background

Natural rubber is an important biopolymer that has been produced for more than a century by cultivating the rubber tree (*Hevea brasiliensis*). Natural rubber obtained by tapping of the rubber trees is used for countless applications, for example for the production of tires, sealings, latex gloves and many, many other items. The main component of rubber latex is the hydrocarbon poly(*cis*-1,4-isoprene). Chemosynthetic rubber is also produced at a scale that is almost comparable to that of the natural compound.

Despite the economic importance of rubber and the enormous amounts of rubber waste materials that are permanently released into the environment, complete degradation in nature is rarely detected and wastes continue to accumulate. Knowledge of the reasons for this is limited. In fact, application is made of this extremely slow natural degradation for example in the use of rubber tyres to provide attachment sites for creating artificial coral reefs. However, microorganisms that can attack rubber have been detected in many ecosystems in which the physical parameters (temperature, pH, salinity) are moderate [1–7]. It is also well known that the initial microbial attack on rubber depends on the ability to produce and secrete rubber-cleaving enzymes into the environment. Only two types of rubber-cleaving enzymes are known. One is the rubber oxygenase RoxA that was first isolated from *Xanthomonas* sp. 35Y [8, 9] and so far has been found only in Gram-negative bacteria [10]. RoxA of *Xanthomonas* sp. 35Y is a *c*-type dihaem dioxygenase and cleaves poly(*cis*-1,4-isoprene) into a C_15_ compound with a terminal keto and aldehyde group (12-oxo-4,8-dimethyl-trideca-4,8-diene-1-al, ODTD) as the main product [11–13]. The other rubber cleaving enzyme is a protein designated as latex clearing protein (Lcp) [1]. It shares no significant sequence homology with RoxA, with cytochrome *c* peroxidases or with dihaeme 7,10-diol synthases [14] and is present in Gram-positive rubber degrading bacteria such as *Streptomyces* sp. K30 [1] and other *Actinobacteria. G. polyisoprenivorans* VH2 and *Streptomyces* sp. K30, two well-studied Gram-positive rubber degraders, oxidatively cleave poly(*cis*-1,4-isoprene) to products of different sizes but with the same keto and aldehyde end groups as in RoxA-generated ODTD [15–17]. There have been different reports published for the co-factor and metal-contents of the Lcps from *Streptomyces* sp. K30 and of *G. polyisoprenivorans* VH2 [15, 17, 18], and at present there are currently only two biochemically characterized Lcp proteins.

In this study, we used a waste pond at a rubber-processing factory in Thailand as a natural enrichment environment for rubber-degrading microorganisms and a source for the isolation of new rubber degrading strains. Taxonomic analysis revealed that one isolated strain was a member of the genus *Rhodococcus*, a taxon that had not been previously identified as having the ability to utilise rubber as a sole source of carbon and energy but that is well known for its members to have a high potential for the biodegradation of recalcitrant compounds [19]. Biochemical and biophysical characterization of the purified recombinant Lcp protein of *Rhodococcus rhodochrous* strain RPK1 revealed some unexpected properties not previously described for any other rubber-degrading enzyme in addition to properties shared with the two other characterized Lcp proteins.

## Results and discussion

### Taxonomic identification of isolate RPK1

**Fig. 1:**
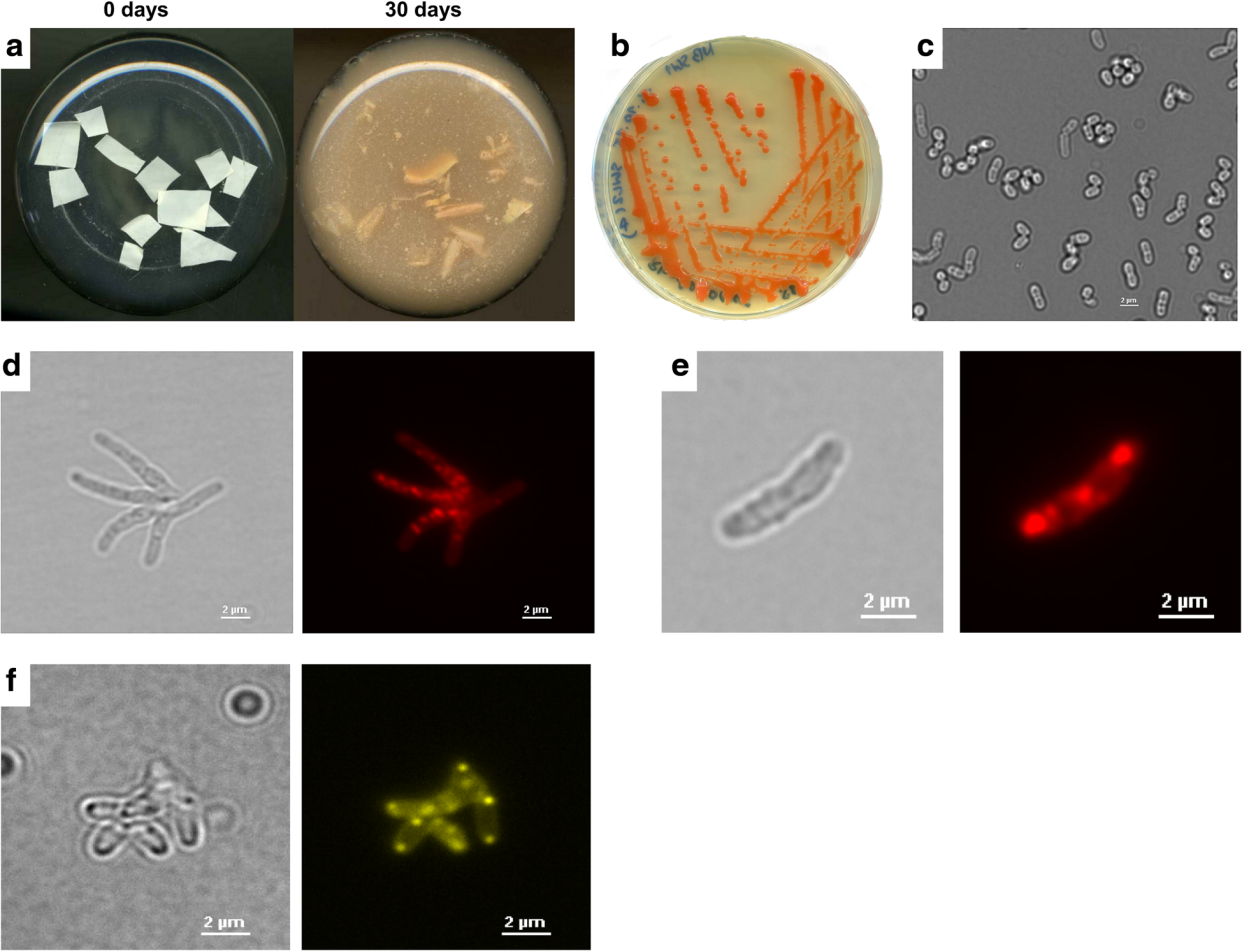
Features of *R. rhodochrous* RPK1. (**a**) Degradation of rubber pieces by *R. rhodochrous* RPK1 after 0 and 30 days of incubation in shaking flasks with mineral salts medium at 30 °C; (**b**) formation of red-coloured colonies of *R. rhodochrous* RPK1 during growth on NB agar; (**c**) morphology of stationary *R. rhodochrous* RPK1 cells in bright field microscopy, note almost coccoid cells; (**d**) *R. rhodochrous* RPK1 cells during growth on NB medium supplemented with acetate (bright field and fluorescent image stained with Nile red). Note, star-like branched cells typical for *R. Rhodochrous*; (**e**) *R. rhodochrous* RPK1 cells during growth on NB medium supplemented with acetate (bright field and fluorescent image stained with Nile red, note, presence of Nile-red-stainable granules, possibly representing PHB granules or triacylglycerol bodies; (**f**) *R. rhodochrous* RPK1 cells during growth on NB medium supplemented with acetate (bright field and fluorescent image stained with DAPI and examined for presence of polyphosphate granules using DAPI-polyphosphate-specific emission filters). Note, presence of cell-pole localized polyphosphate granules in most cells

Isolate RPK1 had a high rubber-degrading activity compared to other rubber degraders in liquid culture, as revealed by pronounced disintegration of rubber pieces (Fig. 1a). However, isolate RPK1 did not form clearing zones on an opaque polyisoprene latex mineral salts agar while known clear zone formers such as *Xanthomonas* sp. 35Y [8] or *Streptomyces coelicolor* strain 1A [3] formed large clearing zones. Isolate RPK1 developed colonies with an intense red colour upon growth and prolonged incubation on NB agar (Fig. 1b). Microscopic examination revealed non-motile cells. Depending on the growth phase the cells were coccoid (cells from late stationary phase), rod-shaped (cells from early and late log phase) or long rods (up to 1 ×5 μm), partially branched and star-like in exponentially growing cultures (Fig. 1c-e). Isolate RPK1 was catalase positive and Gram-positive. It grew well at 43 °C but no colonies developed at 45 °C. Strain RPK1 tolerated the presence of 3 % NaCl (in NB). It accumulated storage compounds that were stainable by Nile red (polyhydroxyalkanoates or triacylglycerols) and strain RPK1 synthesised polyphosphate granules as shown by staining with DAPI (4’,6-diamidine-2-phenylindole) and the use of DAPI-polyphosphate-specific emission filters in fluorescence microscopy (Fig. 1e, f). Isolate RPK1 utilised complex media (NB, LB medium) and grew with mineral salts media containing D-mannitol, fructose, acetate, benzoate or octane as a single carbon source. Glucose, sucrose, gluconate, pentane, petroleum or pyridine (excluding *Rhodococcus pyridinivorans*) were not used for growth. Polymers such as polyhydroxybutyrate (PHB), casein or starch were also not utilised by RPK1. These characteristics, in combination with the red colour of the colonies and the variable morphology of the cells indicated that the isolate RPK1 could be a member of the genus *Rhodococcus*. To verify this assumption we determined the DNA-sequence of the PCR-amplified 16S rRNA gene (accession No KU140418) and compared the sequences to the database by BLAST search. The 16S rRNA gene was 99.7 and 99.2 % identical to *Rhodococcus* MK3027 and to *R. rhodochrous* MTCC11081, respectively. Together with the biochemical and morphological data we concluded that isolate RPK1 is a member of the species *R. rhodochrous*. It differed from the rubber degrading *Xanthomonas* sp. 35Y [8, 9], *Streptomyces* sp. K30 [1], and other rubber degrading streptomycetes [3] by its inability to produce clearing zones on opaque polyisoprene latex agar plates. Previously, bacteria with a strong rubber-degrading activity but with no ability to form clearing zones had been isolated and identified as *Gordonia polyisoprenivorans* or *Gordonia westfalica* [20].

### Identification of the gene coding for the latex clearing protein in *R. rhodochrous* strain RPK1

BLAST analysis revealed that many *Actinobacteria* and all known rubber-degrading *Actinobacteria* for which the genome sequences have been determined have at least one gene that codes for a so-called latex clearing protein (*lcp*) that is suspected to be responsible for the initial oxidative attack on the polyisoprene carbon backbone [1, 21–23]. Remarkably, non clearing zone formers such as *G. polyisoprenivorans* also have functional *lcp* genes [20]. This indicates that the Lcp protein apparently is not directly responsible for the formation of clearing zones during growth on opaque latex-agar. Alignment of the amino acid sequences of the Lcp proteins from different species (most of them annotated as Lcp protein but without verified function or biochemical characterization) revealed conserved regions within the Lcp amino acid sequences [15]. We identified a hypothetical *lcp* gene in the genomes of *R. rhodochrous* strain MTCC11081, *Rhodococcus* sp. MK3027 and *Rhodococcus* sp. ARG-BN062 by screening of the published genome sequences for the presence of *lcp*-like sequences. The deduced amino acid sequence of these hypothetical Lcp proteins included the DUF2236 domain that constitutes the central part of most if not all Lcp proteins [15]. Two oligonucleotides based on the upstream and downstream regions of the *lcp* genes of these *Rhodococcus* strains were generated (Lcp_Rr_-PstI_for and Lcp_Rr_-HindIII_rev) and a PCR reaction was performed with the chromosomal DNA of *R. rhodochrous* strain RPK1. A 1.5 kbp DNA fragment was obtained and its DNA sequence was determined (accession number KU140417). Analysis of the DNA sequence revealed one large open reading frame of 1227 bp that coded for a peptide of 408 amino acids (45.2 kDa, Table 1). The deduced amino acid sequence revealed strong similarities to the postulated Lcp proteins of *R. rhodochrous* strains and of several other *Rhodococcus* sp. strains (81 to 99 % identical amino acids). High degrees of similarities were also detected to many other genome-deduced sequences of putative Lcp proteins from bacteria including those from many streptomycetes and other *Actinobacteria*. When we compared the overall amino acid sequence of Lcp_Rr_ with that of the only two other biochemically characterized Lcp proteins, a 70 % (76 %) identity (similarity) and a 57 % (66 %) identity (similarity) was determined to the Lcp_VH2_ from *G. polyisoprenivorans* [20] and Lcp_K30_ protein from *Streptomyces* sp. K30 [1], respectively (Additional file 1). A 30 amino acid long sequence at the N-terminus of Lcp_Rr_ was predicted to code for a signal peptide that enabled the secretion of the protein. The molecular mass of the predicted mature protein amounted to 42.2 kDa (Table 1).

**Table 1:** Properties of biochemically characterized rubber oxygenases

	Lcp_Rr_	Lcp_K30_	Lcp_VH2_^a^	RoxA_Xsp_
Gene length [bp]	1227	1224	1224	2037
Residues (pre-/mature enzyme)	408/378	407/377	407/371^a^	678/648
Molecular mass (apo-/mature protein) [kDa]	45.2/42.2	44.0/41.0	45.5/41.7^a^	74.7/71.5
strep tagged [kDa]	44.5	43.3		
pH optimum	8	7	7	7
Molar extinction coefficient [10^4^ M^−1^ cm^−1^]	9.5 (407 nm)	8.0 (412 nm)	n.d.	20.6 (406 nm)
Metal atoms per protein molecule				
Fe	0.98	1.16/1.05	n.d.	2.3
Cu	0.36	0.056/-	sub-stoichiometric	-
Mn	-	-	n.d.	-
Ni	-	0.12/0.05	n.d.	-
Zn	-	-	n.d.	-
Mg, Ca	-	n.d.	n.d.	n.d.
Haeme type	*b*-type	*b*-type	not known/no haeme	*c*-type
Oxidation state of haeme iron	Fe^3+^	Fe^3+^		Fe^3+^---O2^-^
UVvis effect upon addition of CO	no	no		yes
Specific activity [U/mg] (23/30/37 °C)	0.9/3.1/^b^	1.5/n.d./4.6	1.3/n.d./n.d.	n.d./0.48/n.d.
Melting point	n.d.	61.5 °C	n.d.	54.3 °C
Conserved DUF2236 residues^c^				
R	163	164	161	
T	167	168	165	
H	197	198	195	

*n.d* not determined

*-* not detectable (below detection limit)

^(a)^ deduced from [8, 9, 15] and SignalP4.1 Server

^(b)^ activities of Lcp_Rr_ at 37 °C were not reproducible in oxygen consumption assay

^(c)^ position in native sequence

### Expression and purification of Lcp_Rr_

**Fig. 2:**
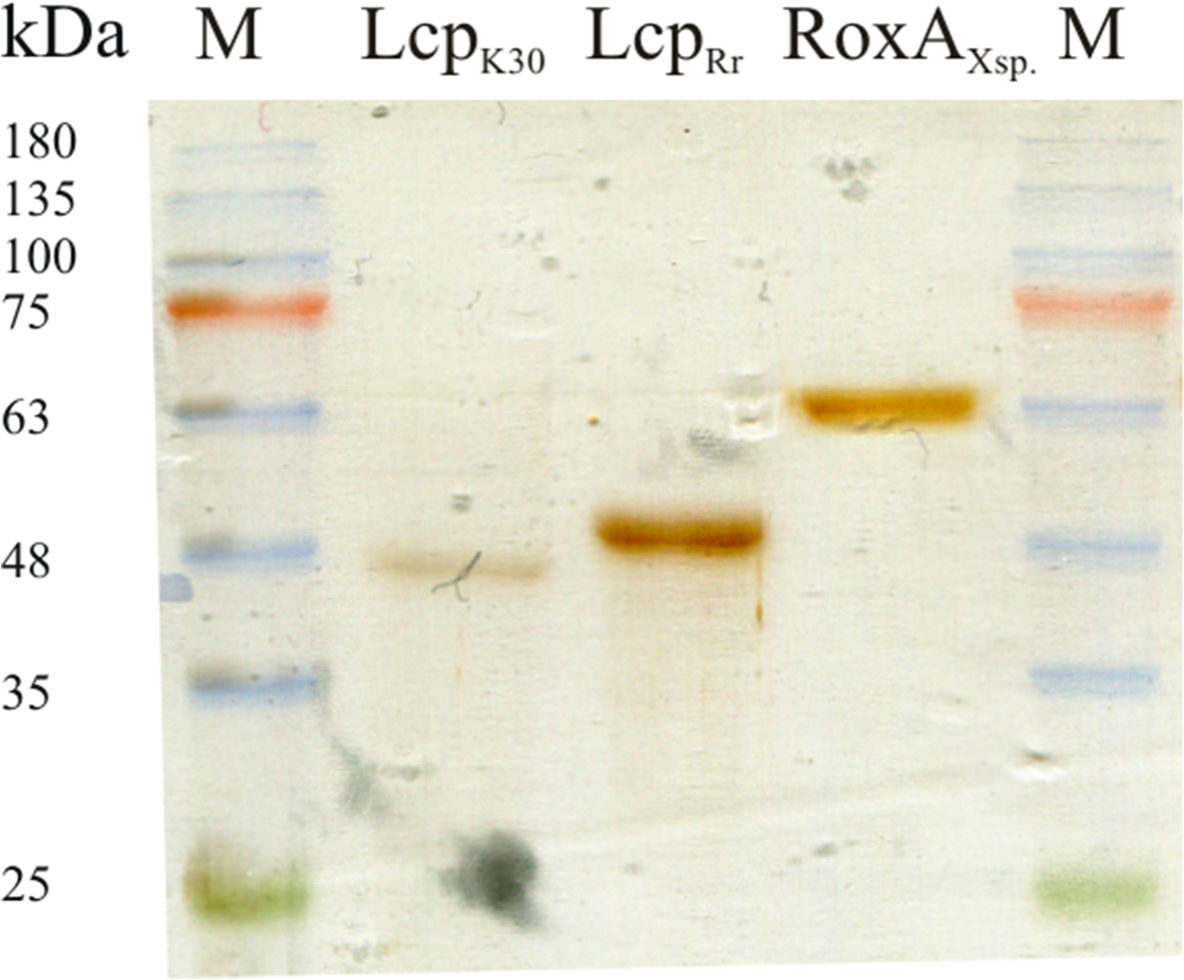
SDS-PAGE of purified Lcp_K30_, Lcp_Rr_ and RoxA_Xsp_. Purified proteins were separated by reducing SDS-PAGE and subsequently stained with silver. The kDa values of marker proteins (M) are indicated

The DNA sequence coding for the Lcp_Rr_ signal peptide was replaced by a Strep-tag coding sequence and the modified gene was cloned under the control of an L-rhamnose-dependent promoter into p4782.1 and subsequently transformed to *E. coli* JM109. The Lcp_Rr_ protein was purified from the combined cells of a 4.8 L *E. coli* (p4782.1::*lcp*_*Rr*_) culture as described in the method section. A yield of approximately 7.7 mg of purified Lcp_Rr_ protein (5.3 mg/mL in BCA assay) was obtained after the final purification step. The Lcp proteins of S*treptomyces* sp. K30 (Lcp_K30_) and of the rubber oxygenase RoxA of *Xanthomonas* sp. strain 35Y (RoxA_Xsp_) were also purified and were used for comparison purposes. All purified proteins were separated by SDS-PAGE and checked for purity. As shown in Fig. 2, the Lcp_Rr_, Lcp_K30_ and RoxA_Xsp_ proteins were almost homogenous. Both Lcp_Rr_, and Lcp_K30_ proteins migrated at a slightly higher apparent molecular mass (50 and 47 kDa) as deduced from the gene sequences (44.5 and 43.3 kDa, respectively). Most remarkably, concentrated Lcp_Rr_ had a brownish colour. This was in sharp contrast to the red colour of the concentrated solutions of Lcp_K30_ or of RoxA_Xsp_ and indicated that there were substantial differences of the Lcp_Rr_ protein in comparison to the other rubber-cleaving enzymes.

### Biochemical properties of Lcp_Rr_

**Fig. 3:**
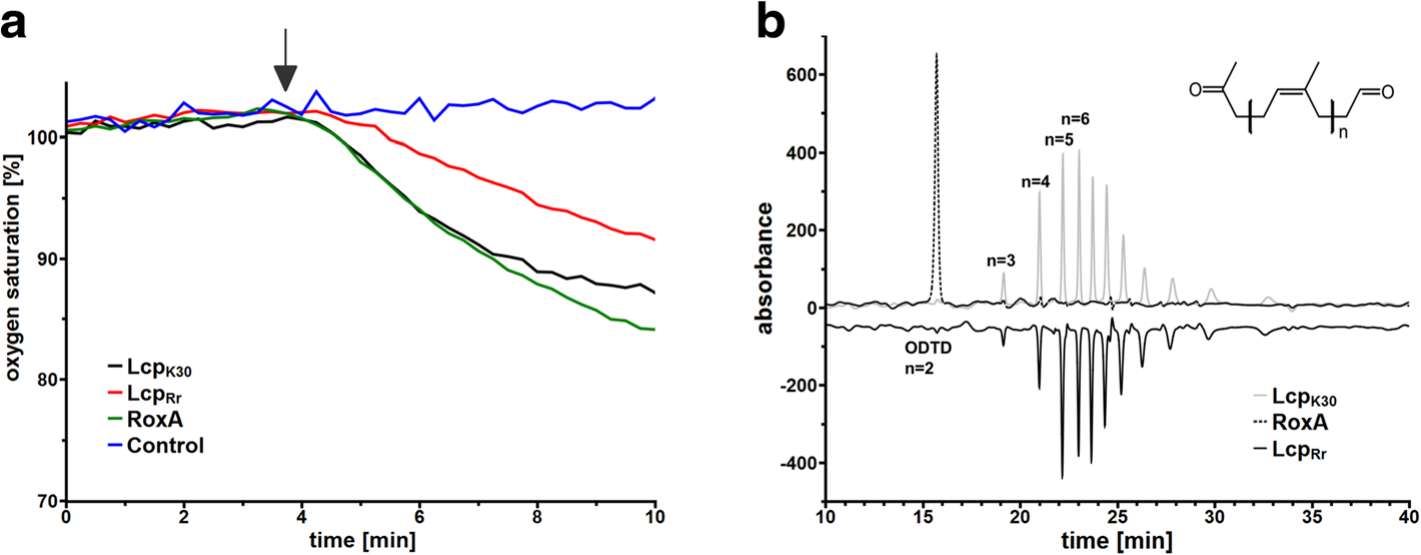
Assays for oxidative polyisoprene cleavage. (**a**) Oxygen consumption assay: 4 μg of the indicated enzyme (control without enzyme) was added after 3.75 min (arrow) and oxygen saturation was recorded. Activities were calculated from the slopes of the graphs during the first minutes. The mixtures were extracted with ethylacetate after 90 min of incubation at 23 °C and the formed cleavage products were analysed by HPLC (**b**). ODTD was the main cleavage product of RoxA_Xsp_ but was present only in trace amounts in Lcp_Rr_ and Lcp_K30_ preparations. The graph for Lcp_Rr_ is given in its inverse orientation. Assays with each enzyme were performed at least three times; one typical dataset is shown

**Fig. 4:**
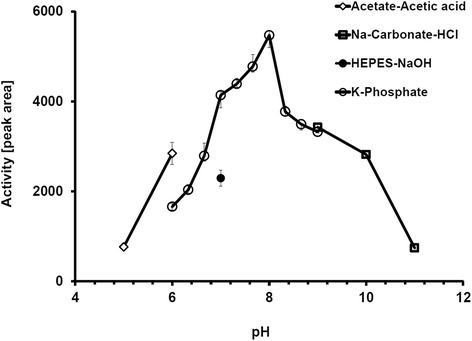
pH optimum of Lcp_Rr_. The pH optimum was determined using the HPLC-based product assay in a pH range of 5 to 11 using acetate buffer (pH 5 - pH 6, diamonds), phosphate buffer (pH 6 - pH 9, open circles), carbonate buffer (pH 9 – pH 11, squares), or HEPES (pH 7, closed circle). Assays were performed with two biological and two technical replicates. Error bars indicate standard deviation

**Fig. 5:**
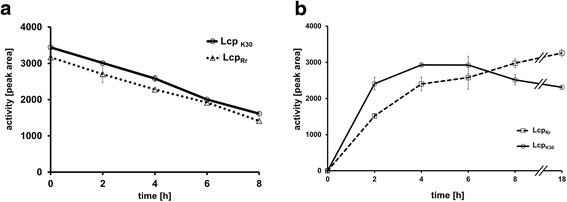
Stability of Lcp_Rr_ and Lcp_Rr_ and product formation. Lcp proteins were incubated in the presence of polyisoprene latex for 0 to 8 h at room temperature and the amount of formed products was determined by HPLC (**a**). Lcp proteins were incubated at 37 °C for up to 18 h before the standard activity assay was performed (**b**). Assays were performed with two biological and two technical replicates

The purified Lcp_Rr_ protein was tested for its rubber cleaving activity using both the oxygen consumption and the HPLC-based rubber cleavage product assay. The oxygen consumption assay (Fig. 3a) confirmed that Lcp_Rr_ cleaved poly(*cis*-1,4-isoprene) latex in an oxygen-dependent manner; specific activities of 0.9 U/mg and of 3.1 U/mg were determined for Lcp_Rr_ at pH 8 and at 23 °C and 30 °C, respectively (Table 1). Variable data were determined for the specific activity of Lcp_Rr_ at 37 °C possibly because of the decreasing stability of the Lcp_Rr_ protein at higher temperatures (see below). The HPLC (Fig. 3b) and Fuchsin assay (Additional file 2) revealed that Lcp_Rr_ produced the same mixture of polyisoprene cleavage products (C_20_ and higher oligo-isoprenoids with terminal keto and aldehyde groups) that had been determined for Lcp_K30_. ODTD was only detectable in trace amounts for Lcp_Rr_ or for Lcp_K30_ but was the main product of the RoxA_Xsp_-derived rubber cleavage products. Determination of the activities of purified Lcp_Rr_ at different pH values using the HPLC-based product assay revealed a pH optimum of around pH 8 (Fig. 4) that was about one pH unit higher than the pH optimum that had been previously determined for RoxA_Xsp_ or for Lcp_K30_ and Lcp1_VH2_ [9, 15, 17]. The stability of all the Lcp preparations decreased upon incubation in buffer at 37 °C (Fig. 5a). In accordance with this, the concentration of rubber degrading products in an *in vitro* latex cleavage assay with Lcp_Rr_ or Lcp_K30_ increased for only 4–8 h (Fig. 5b). RoxA_Xsp_, on the other hand, was much more stable and continuously produced ODTD molecules for up to 70 h [24].

### Lcp_Rr_ is a *b*–type cytochrome and revealed remarkable differences to Lcp_K30_

**Fig. 6:**
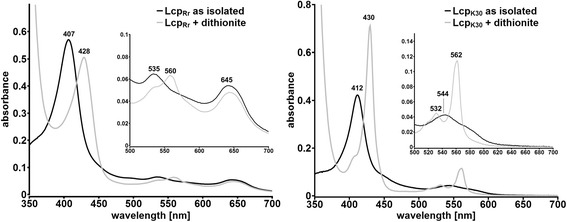
UVvis spectrum of Lcp_Rr_ and Lcp_K30_ *as isolated* (black lines) and after reduction with dithionite (grey lines). Both Lcp proteins show a prominent band at 407 (412 nm in case of Lcp_K30_) that is characteristic for porphyrines. After reduction with dithionite a characteristic shift of the α-band to 428 (430) nm as well as an increase in the Q-band region (560/562 nm) was observable. Assays were repeated at least three times with two separate protein batches. A typical experiment is shown

Concentrated solutions of Lcp_Rr_ had a brown colour while the Lcp_K30_ solutions were red. Figure. 6 shows a comparison of the UVvis spectra of the purified Lcp_K30_ and Lcp_Rr_ proteins in the *as isolated* (oxidised) and in the dithionite-reduced state. Lcp_Rr_ (and Lcp_K30_) *as isolated* both showed similar absorption maxima at 407 (412) nm and at 535 (544) nm that are typical for haem-containing proteins in the oxidised form. However, purified Lcp_Rr_ had an additional broad absorption maximum around 645 nm. The absorption band at 645 nm was absent in Lcp_K30_ and in other biochemically characterized RoxA proteins such as RoxA_Xsp_ and RoxA_Cco_ [10] and was responsible for the different (brown) colour of Lcp_Rr_. When the Lcp preparations were chemically reduced by the addition of sodium dithionite, the absorption bands at 407 (412) nm and 535 (544) nm shifted to 428 (430) nm and 560 (562) nm. A comparison of the reduced spectra of both Lcp proteins showed differences in the Q-bands (500 – 600 nm). Apparently, Lcp_Rr_ is far less pronounced in this region than Lcp_K30_. Nevertheless, these data corresponded to the Soret and Q-bands that are typical for haem-containing proteins and strongly indicated that Lcp_Rr_ is a haem-containing protein. The band around 645 nm, however, was not changed by the addition of dithionite.

To confirm that Lcp_Rr_ is a haem-containing protein and to determine its haem type, a metal analysis and a spectral analysis by the haem-bipyridyl assay were performed. 6.5 μg Fe/mL Lcp_Rr_ protein solution (5.3 mg protein/mL) were determined. This corresponded almost perfectly with a stoichiometry of one atom Fe per one Lcp_Rr_ molecule. It was of interest that low amounts of copper (2.8 μg/mL) were also identified and corresponded to 0.36 atoms Cu per one Lcp_Rr_ molecule. Zinc was detected at the detection limit (0.1 μg/mL) and Nickel was below the detection limit (<0.1 μg/mL); other metals (vanadium to zinc tested) were also not detected in significant amounts. Divalent cations such as magnesium or calcium were not present (below the detection limit of 0.1 μg/mL) (Table 1). The presence of approximately one third of an atom% Cu per Lcp_Rr_ molecule was unexpected because only traces of copper had been previously detected in Lcp_K30_ [18]. The determined amount of copper in Lcp_Rr_, however, was too high to be explained by an error in the determination of the metal or protein concentration. One possibility could be that the amount of copper was due to a contamination of the protein by traces of copper present in either the growth medium or in the buffer ingredients. For example, the used batch of NaCl that was present in some purification buffers was only of 98 % purity and could contain traces of heavy metals. However, sub-stoichiometric amounts of copper (precise concentration not known) had been also detected in Lcp1_VH2_ of *G. polyisoprenivorans* [15]. Addition of an equimolar concentration or of a 10-fold molar excess of copper ions [Cu(II)Cl_2_] to the assay mixture with purified Lcp_Rr_ had no detectable effect on the UVvis spectrum or on the activity of Lcp_Rr_. The addition of 50 μM CuSO_4_ to the Lcp_Rr_-expression culture produced no increased activity or yield of Lcp_Rr_. At present, there is no convincing explanation for the finding of variable sub-stoichiometric amounts of copper in the purified Lcp proteins from *R. rhodochrous* RPK1.

An absorption maximum of 556 nm was determined using the bipyridyl assay for Lcp_Rr_ and for haemoglobin that was used as a *b*-type cytochrome control protein (Additional file 3). This result indicated the presence of a *b-*type haem in Lcp_Rr_. In contrast to the covalently linked *c*-type cytochromes, the haem groups of the *b*-type cytochromes are not covalently linked to the peptide chain and can be therefore extracted by an acid solvent extraction [25]. Acid solvent extraction of the purified Lcp_Rr_ yielded a coloured supernatant and a non-coloured precipitate. In contrast, solvent extraction of the *c*-type cytochromes such as RoxA_Xsp_ or of other commercially available cytochrome *c* enzymes yielded a non-coloured supernatant and a red precipitate which is in agreement with the covalent attachment of porphyrin to the polypeptide. MALDI-ToF analysis of the purified Lcp_Rr_ resulted in the identification of ions with *m/z* values of 616 (data not shown) which is typical for haem *b* [26]. Taken together, all these results indicated that Lcp_Rr_ is a *b*-type cytochrome similar to Lcp_K30_ [18] Notably, MALDI-ToF analysis of Lcp_Rr_ also revealed an ion species with *m/z* values of 619 besides that of 616 which could correspond to a verdo-haem [27]. As the activity of purified Lcp_Rr_ rapidly and substantially decreased during storage, the haem species with *m/z* value of 619 could represent a haem degradation product of the inactivated Lcp_Rr_.

### Lcp_Rr_ is insensitive to most chelating inhibitors

**Fig. 7:**
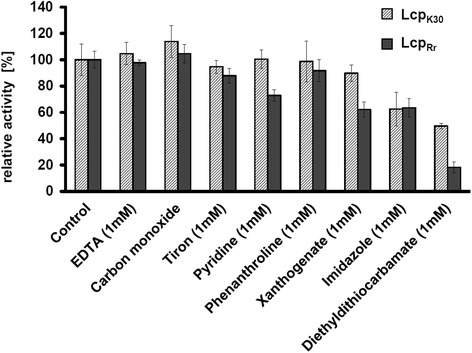
Inhibition of Lcp_Rr_ by potential inhibitors. Activity was determined using the HPLC-based detection of polyisoprene cleavage products after 2 h of incubation of polyisoprene latex with Lcp at room temperature and subsequent solvent extraction of the products. The final concentration of inhibitors was 1 mM. The 100 % value corresponded to the area of the C_35_ (23 min) product peak (see Fig. 3b). Sensitivity to carbon monoxide was tested by replacing 0.5 volumes of (oxygenated) assay buffer by a deoxygenated and CO-saturated assay buffer. Cleavage products were solvent-extracted after 2 h of incubation time. At least two repetitions with two technical replicates were performed for each inhibitor. Error bars indicate the upper and lower values of one experiment

Metal-dependent proteins are often inhibited by chelating compounds. Therefore, a variety of known chelator compounds was tested for their effects on the activity of Lcp_Rr_ using the HPLC-based activity assay. EDTA, tiron, or phenanthroline had no significant effect on the activity (Fig. 7). Ethyl xanthogenate partially inhibited Lcp_Rr_ by ≈ 40 % similar to that for Lcp_K30_ but was different from the Lcp purified from *G. polyisoprenivorans* (Lcp1_VH2_) that completely inhibited Lcp_VH2_ at 2 mM xanthogenate [15]. The only compound that had a strong effect on the activity of Lcp_Rr_ was the metal chelator diethyl dithiocarbamate (82 % inhibition, Fig. 7). However, diethyl dithiocarbamate had no effect on the UVvis spectrum of Lcp_Rr_ and this excluded a direct effect of the inhibitor at the haem site. Carbon monoxide, that completely inactivated RoxA and led to a prominent band at 415 nm by UVvis spectroscopy [18], had no effect on the absorption spectrum of Lcp_Rr_ or Lcp_K30_*as isolated* and this was in agreement with the presence of an oxidised (Fe^3+^) haem centre. Carbon monoxide had no inhibitory effect on the polyisoprene cleavage during the assay for the HPLC-based product when sufficient oxygen was also present in the assay mixture. However, when Lcp_Rr_ was incubated in carbon monoxide-saturated and oxygen-free buffer before it was added to an oxygenated polyisoprene latex assay solution, a lag phase of Lcp_Rr_ in its ability to consume oxygen was observed. The oxygen consumption and polyisoprene-cleaving activities were recovered within 30 to 50 min of incubation and exposure of the assay solution to air. The same result was obtained when Lcp_K30_ was exposed to carbon monoxide. Addition of carbon monoxide to the dithionite-reduced Lcp_Rr_ or Lcp_K30_ had visible effects on the UVvis spectra as revealed by the increase of the α-band of Lcp_Rr_ and Lcp_K30_. The effect of carbon monoxide on the UVvis spectrum of Lcp was reversible by addition of a dioxygen atmosphere and indicated that the binding of carbon monoxide to the chemically reduced haem group in Lcp was reversible. This is different to RoxA_Xsp_ that binds carbon monoxide more strongly and completely inhibits the activity. An apparent consequence of these data is that the haems of the Lcp proteins undergo a reversible Fe^3+^ to Fe^2+^ reduction during oxidative polyisoprene-cleavage and that the reduced Lcp proteins were the carbon monoxide-sensitive molecular species. This is the first evidence for a switch in the oxidation state of the active haem site of an Lcp protein during catalysis.

### Lcp_Rr_ but not Lcp_K30_ is accessible for external ligands

**Fig. 8:**
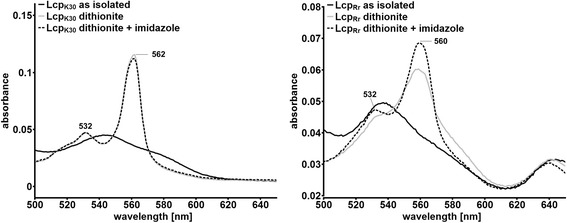
UVvis spectrum of Lcp_Rr_ and Lcp_K30_ before and after the addition of 1 mM imidazole (*as isolated*/after reduction) in the area of the Q-band. No significant change was observable for Lcp_K30_ whereas for Lcp_Rr_ a substantial increase at 560 nm was determined upon addition of imidazole. Assays were repeated two times with two separate protein batches. A typical experiment is shown

Previous studies on rubber oxygenase RoxA had revealed that the active haem site in RoxA had only one axial amino acid ligand. The other axial ligand was a dioxygen molecule that was stably bound to haem in a Fe^3+^----O_2_^−^ transition state. The oxygen molecule in RoxA could be partially removed by the addition of imidazole thereby moving the negative charge from the oxygen molecule to the iron atom (Fe^2+^). This charge transfer resulted in a small visible change of the UVvis spectrum as revealed by an increase of the absorption of the Q-bands at 549 nm [12, 15–17]. When imidazole was added to the dithionite-reduced RoxA, substantial increases in the Soret and Q-bands were determined compared to the reduced RoxA bands without imidazole [18, 24]. This increase in absorption was interpreted as the result of the binding of the imidazole molecule to the (now) free sixth (axial) coordination site of the haem iron. When we performed an analog experiment with purified Lcp_Rr_ and with purified Lcp_K30_ we found remarkable differences between both Lcp proteins: addition of imidazole to the dithionite-reduced Lcp_K30_ had no effect on the UVvis spectrum and there was no detectable increase of the Q-bands. This indicated that the 6th coordination site of the haem apparently was not accessible for imidazole and the Lcp_K30_ protein was present in a “closed state”. Binding of the substrate (polyisoprene) would therefore require a conformational change of the Lcp_K30_ structure. In contrast, addition of imidazole to the dithionite-reduced Lcp_Rr_ protein resulted in a substantial increase of the Soret- and Q-bands (Fig. 8) and this can be explained by the binding of imidazole to the reduced haem. Similar results were obtained when both the Lcp proteins were treated with mercaptoethanol: no change of the UVvis spectrum was determined for Lcp_K30_ while prominent changes were detected for the Lcp_Rr_ protein (Additional file 4). In conclusion, Lcp_K30_ and Lcp_Rr_ seem to rest in a different conformation in their *as isolated* states. While Lcp_K30_*as isolated* was in a six-fold coordinated “closed” state, the haem group of Lcp_Rr_ was readily accessible to external ligands and substrates, and this indicated a five-fold coordinated “open” state. Further evidence for this can be found in the UVvis spectra of five-fold coordinated myoglobin in the oxidised (met myoglobin) and reduced (desoxy-myoglobin) state. The UVvis spectra of the latter proteins showed similarities to the corresponding spectra of Lcp_Rr_, particularly in the region of the less pronounced Q-bands of reduced Lcp_Rr_ compared to Lcp_K30_ as well as in the 645 nm region in the oxidised (*as isolated*) state [28]. The presence of the 645 nm absorption band in Lcp_Rr_ might be also explained by a charge transfer phenomenon of a charged residue/ion in close neighbourhood to the haem group in Lcp_Rr_ and in its absence in Lcp_K30_ [29]. Unfortunately, only the RoxA structure [30] but no Lcp structure was available to obtain direct support for our assumption.

*lcp* genes are frequently present in the genomes of *Actinobacteria* [22] and many rubber degrading species have been described for members from this group [3–5, 23, 31, 32]. Most of the rubber degrading actinomycetes such as *Streptomyces* sp. K30 [1], *Streptomyces coelicolor* 1A, and many others [3, 6, 33] produce clearing zones on opaque polyisoprene latex agar. However several of the most potent rubber degraders do not produce clearing zones and apparently need a close contact to the rubber material they degrade. Two well known rubber degrading *Gordonia* species (*G. polyisoprenivorans* and *G. westfalica*) [20, 22] and also the strain from this study (*R. rhodochrous* RPK1) belong to this group of non-clearing zone formers. One might speculate that the Lcp proteins of non-clearing zone formers constitute a group that have an open conformation with free access to the active site (no conformational change is needed) and that the other Lcp proteins that have an ability to form a clearing zone have a closed form. The prototype of the first group would be Lcp_Rr_ and the prototype of the latter would be Lcp_K30_. It will be necessary to biochemically investigate more Lcp proteins and to solve the structure of Lcp proteins to find evidence for or against this hypothesis.

## Conclusions

This study extends the list of biochemically characterized rubber-degrading non-clearing zone formers by latex clearing proteins (Lcp) to the genus *Rhodococcus* (besides *Gordonia*). The detection of rubber-cleaving activity with purified Lcp_Rr_ and the absence of clearing zones during growth on polyisoprene latex agar raises the question of whether the designation “latex clearing protein” has been well-chosen. Rubber oxygenase B (RoxB) would be an appropriate alternative. However, the designation Lcp has been used is several previous publications and has also been used for many annotated genes in genome-sequenced *Actinobacteria*. Re-classification of Lcp as RoxB therefore could lead to the confusion of other research workers.

The isolation and characterization of the Lcp protein of *R. rhodochrous* RPK1 in this study showed that all the so far studied Lcp proteins can differ in some spectroscopic features and/or in spatial arrangements of their metal ions/cofactors and indicate the presence of two or even more subgroups of Lcp proteins. It will be necessary to study more Lcp proteins to reveal the complete variability of rubber degrading enzymes present in rubber-degrading organisms.

## Methods

### Bacterial strains, plasmids and culture conditions

Table 2 shows the bacterial strains, plasmids and oligonucleotides that were used in this study. *R. rhodochrous* strain RPK1 was grown with nutrient broth (NB) medium or in mineral salts medium (MSM, 9 g/L Na_2_HPO_4_ ×12 H_2_O, 1.5 g/L KH_2_PO_4_, 1 g/L NH_4_NO_3_, 0.2 g/L MgSO_4_ ×7 H_2_O, 0.02 g/L CaCl_2_ ×2 H_2_O, 1.2 mg/L Fe(III)ammonium citrate with solid rubber pieces or with water-soluble carbon sources as indicated at 30 °C. Pieces (1 cm ×1 cm) of heat-sterilised vulcanised rubber (commercial but not-powdered rubber protecting gloves) were added to the sterile mineral salts medium during the enrichment and growth of *R. rhodochrous* on rubber (0.6 % [wt/vol]). Plasmid-carrying recombinant *E. coli* strains were grown with LB medium at 22 °C or 37 °C in the presence of the appropriate antibiotic (ampicillin or kanamycin). Polyisoprene latex was kindly provided by Weber and Schaer, Hamburg (Germany) and was used after 3 washing steps in 0.1 % (wt/vol) Nonidet P40. For purification of Lcp_Rr_, recombinan*t E. coli* cells were grown in LB medium supplemented with 0.1 % (wt/vol) L-rhamnose at 22 °C. Utilization of carbon sources was tested on mineral salts agar with separately filter-sterilised carbon sources at the following end concentrations (sugars, sugar alcohols and sugar acids at 0.5 % [wt/vol], sodium acetate [0.25 %, wt/vol], sodium benzoate [0.1 %, wt/vol]). Volatile compounds (alkanes) were applied by adding a quantity of 100 μL to a sterile filter paper placed in the lid of a petri disk. The plates were sealed with parafilm and incubated separately at 30 °C. Utilization of PHB was tested on PHB overlay plates as described previously [34]. Growth at different temperatures was tested on NB agar plates.

**Table 2:** Bacterial strains, plasmids and oligonucleotides used in this study

Strain or plasmid	Relevant characteristics	Reference
*E. coli* JM109	Plasmid storage and expression of *lcp*	
*E. coli* XL1-blue	Transformation strain	Stratagene
*Rhodococcus rhodochrous* RPK1	Wild type strain, degrades rubber	this study
pUC9::*strep*-*lcp*_*K30*_ (SN5339)	Cloning vector for *lcp*_*K30*_, Ap^r^	[18]
pUC9::*strep*-*lcp*_*Rr*_ (SN5759)	Cloning vector for *lcp*_*Rr*_, Ap^r^	this study
p4782.1 (SN3513)	Mobilizable broad host range	[37]
	Expression vector, Km^r^	
p4782.1::*strep-lcp*_*K30*_ (SN5496)	Coding sequence of *strep-lcp*_*K30*_ under	[18]
	Rhamnose promoter control, Km^r^	
p4782.1::*strep-lcp*_*Rr*_ (SN5760)	Coding sequence of *strep-lcp*_*Rr*_ under	this study
	Rhamnose promoter control, Km^r^	
Oligonucleotides		
Lcp_Rr-_complete_for	GCAGAATCCACATGTCCT	
Lcp_Rr-_complete_rev	CGACAAACCCACAGATGA	
Lcp_Rr_-mature-PstI_for	GGGCCTGCAGCGGCCCTGGAGGTGGTCGCC	
Lcp_Rr_-mature-HindIII_rev	CCGGTAAGCTTTCAGGGATAGTTGGG	
16S-universal-for	GAGTTTGATC(A/C)TGGCTCAG	
16S-universal-rev	GG(C/T)TACCTTGTTACGACT	
16S-Rr-complete_for	CTGGCGCGGTGCTTAAC	
16S-Rr-complete_rev	CAGTAATTCCGGACAACG	

Kanamycin resistance (Km^r^), Ampicillin resistance (Ap^r^)

### Enrichment and isolation of rubber-degrading microorganisms

Liquid from a waste pond at a rubber latex processing factory in Thailand (Namom rubber factory at Namom, Songkhla) was used as an inoculum to enrich for rubber-degrading microorganisms in a mineral salts medium (MSM) that had been supplemented with 1×1 cm pieces of rubber gloves as a sole source of carbon and energy. After two weeks of incubation at 30 °C, 0.1 volumes (without pieces of rubber) were transferred to fresh medium and incubated for an additional month. Substantial disintegration of the new rubber pieces became visible and indicated that active rubber-degrading microorganisms were present. Several bacterial strains were isolated from this enrichment culture by repeated purification from streaks onto NB and LB agar plates. Each isolate was subsequently tested for its ability to degrade rubber in liquid MSM with rubber pieces as carbon source. One isolate (designated as isolate RPK1) with strong rubber-degrading activity was selected for this study.

### Cloning and heterologous expression of *lcp*_*Rr*_, and determination of the 16S rRNA gene sequence of isolate RPK1

The *lcp*_***Rr***_ gene was amplified using the chromosomal DNA from *R. rhodochrous* strain RPK1 as template and the oligonucleotides Lcp_Rr_-complete_for and Lcp_Rr_-complete_rev as PCR primers and Takara Primestar DNA polymerase as the proof-reading polymerizing enzyme. The DNA sequence of the product was determined and is available under the accession no KU140417. Alternatively, the coding sequence of mature Lcp_Rr_ was amplified from chromosomal DNA using Lcp_Rr_-mature-*Pst*I_for and Lcp_Rr_-mature-*Hind*III_rev as primers. The DNA products were purified via agarose gel electrophoresis, cleaved with restriction enzymes *Pst*I and *Hind*III and ligated into plasmid pUC9::*lcp*_*K30*_ that had been cleaved by the same restriction enzymes. The coding sequence for strep-tagged *lcp*_*R*r_ was cut out using *Hind*III and *Nde*I and was subsequently ligated into the expression plasmid p4782.1 and transformed to competent *E. coli* JM109 cells.

A part of the 16S rRNA gene of the isolate RPK1 was PCR-amplified using the primers 16S-universal_for and 16S-universal_rev. The DNA sequence of the resulting PCR product was determined (1412 bp) and revealed a strong similarity to the 16S rRNA genes of several *Rhodococcus* sp. strains. The 16S rRNA gene sequence of isolate RPK1 was determined after PCR amplification using the primers (16S-Rr-complete_for and 16S-Rr-complete_rev), that were specific for the ends of the known 16S rRNA gene sequences of *R. rhodochrous* strains taken form the NCBI data base, and is now available under the accession no KU140418.

### Purification of Lcp_Rr_, Lcp_K30_ and of RoxA_Xsp_

Purification of the rubber oxygenase of *Xanthomonas* sp. 35Y (RoxA_Xsp_) and latex clearing protein Lcp_K30_ was performed as described previously [12, 18] Lcp_Rr_ was purified as follows: eight individual 600 mL LB cultures in 3 litre Erlenmeyer flasks were inoculated each with 0.02 volumes of a seed culture of *E. coli* JM109 harbouring the plasmid p4782.1::*lcp*_*Rr*_ that had been grown with the same medium. It was important that L-rhamnose (0.1 %, wt/vol) was present right from the beginning in the main cultures to maximise the yield of the expressed Lcp_Rr_ protein. Cells of the main culture were harvested by centrifugation after ≈ 20 h of growth at 22 °C and were immediately used for protein purification. The cell pellet was resuspended in 100 mM potassium phosphate buffer, pH 7.7, containing 150 mM sodium chloride (KPN, 2 mL KPN/g cell wet weight). A soluble cell extract was prepared by two French press steps and subsequent centrifugation at 40,000 *g* for 40 min. The supernatant (≈ 60 mL) was directly applied to a 10 mL Strep-Tactin HC gravity flow column that had been equilibrated with KPN buffer. The column was washed with at least five volumes of KPN buffer before the Lcp_Rr_ protein was eluted by ≈ 30 mL of 5 mM desthiobiotin dissolved in KPN. Lcp_Rr_-containing fractions were combined, desalted by running through a G25 Sephadex (26/160) Hiprep desalting column (53 mL bed volume) that had been equilibrated with 1 mM potassium phosphate (KP) buffer, pH 7.0 and subsequently concentrated to 1–2 mL via ultrafiltration (10 kDa cut-off). Remaining impurities were removed by chromatography on a Superdex 200 column (16/600, equilibrated with 1 mM KP, pH 7) at a flow rate of 1 mL/min. Combined Lcp_Rr_-containing fractions were ultrafiltrated (10 kDa cut-off) and concentrated to ≈ 1.5 mL. Aliquots of the purified Lcp_Rr_ protein were stored on ice for about 3 days (Lcp_K30_ up to 1 week) or shock-frozen with liquid nitrogen and stored at -70 °C.

### Determination of the cytochrome type of Lcp_Rr_

The haem type of Lcp_Rr_ was determined by the bi-pyridyl assay as described elsewhere [35]. Purified RoxA_Xsp_, cytochrome *c* (horseheart, type III, Sigma, St. Louis, USA) (both *c*-type cytochromes) and haemoglobin (*b*-type) (bovine, Sigma, St. Louis, USA) were used as controls for known *c*-type and *b*-type cytochromes, respectively. 25 μL of the respective protein stock solution (4–8 mg/mL) was added to 975 μL solution A (100 mM sodium hydroxide, 20 % (v/v) pyridine, 0.3 mM potassium ferricyanide). Subsequently, 2–5 mg sodium dithionite were added and the spectrum of the reduced cytochrome was recorded. The absorption maxima of the resulting α-bands were characteristic for *b*-type (556 nm) and *c*-type (550 nm) cytochromes. Bi-pyridyl-haem complexes of *α*-type cytochromes absorb at 584–588 nm. Additional assays for determination of the haem type were performed via extraction of haem by acidic acetone and by a matrix assisted laser desorption ionization time of flight (MALDI-ToF) analysis as previously described in detail [18].

### Assay of Lcp activity

An HPLC-based assay for Lcp_Rr_-derived polyisoprene degradation products was used for most routine assays: poly(*cis*-1,4-isoprene) latex was diluted with 100 mM KP buffer, pH 7, to 0.2 % (assay volume 0.7 mL) and incubated in the presence of purified Lcp protein for 2 h at a temperature as indicated (for routine assay at room temperature [23 °C]). In the case of inhibition studies, the corresponding compound was added and gently solubilised in the reaction mix before the enzyme was added (final inhibitor concentration 1 mM). The products were extracted with 1 mL ethyl acetate (in a 2 mL Eppendorf tube), dried, and dissolved in 100 μL methanol. Aliquots were applied to an RP8 HPLC column (12 ×4 mm, 5 μm particle size, 0.7 mL/min) with water (A) and methanol (B) as mobile phases. The concentration of B was increased from 50 % (v/v) to 100 % (v/v) within 15 min; products were detected at 210 nm. The C_35_ product peak (at ≈ 23 min) was used for quantification and compared to a control without inhibitor. Alternatively, activity of Lcp_Rr_ was assayed by determination of the rate of oxygen consumption in an OXY-4 mini apparatus (PreSens, Regensburg, Germany) as described previously [18]. Triplicates and controls without Lcp_Rr_ or with heat-inactivated Lcp_Rr_ were recorded simultaneously. A stability assay was performed by incubation of the purified Lcp_Rr_ protein in the assay buffer at 37 °C for variable time periods. The remaining activity of the protein was determined as described above.

### Other techniques

The concentration of protein solutions was determined by the bicinchoninic acid (BCA) method. The concentrations of purified rubber-cleaving enzymes were also determined from the molar extinction coefficients of Lcp_Rr_, Lcp_K30_ and RoxA_Xsp_: Lcp_Rr_, ε_407_ = 9.5 10^4^ M^−1^ cm^−1^, Lcp_K30_, ε_412_ = 8.0 10^4^ M^−1^ cm^−1^, and RoxA_Xsp_, ε_406_ = 2.06 ×10^5^ M^−1^ cm^−1^. Separation of proteins was performed by polyacrylamide gel electrophoresis in the presence of sodium dodecyl sulphate (SDS-PAGE) under reducing (2-mercaptoethanol) conditions. The metal content of the purified Lcp protein was determined using inductively coupled plasma-MS (ICP-MS) by the Spuren-Analytisches Laboratorium Dr. Baumann (Germany). Fuchsin staining of polyisoprene degradation products was performed by addition of a 1 % Fuchsin solution (0.5 g Fuchsin, 12.5 mL acetic acid, 2.5 g Na_2_S_2_O_3_, 0.2 mL HCl (37 %) and 37.5 mL H_2_O) to the Lcp assay mixture. Staining of the cells for PHB and polyphosphate was performed as described previously [36].

### Ethics approval

Not applicable.

### Consent for publication

Not applicable.

### Availability of data and materials

The DNA sequence of the *R. rhodochrous* RPK1 *lcp* and 16S rRNA gene have been submitted to NCBI and are available under the accession No KU140417 and KU140418. The *R. rhodochrous* RPK1 strain has been deposited at the Deutsche Sammlung für Mikroorganismen und Zellkulturen (DSMZ) and can be obtained once the Nagoya protocol regulations have been finished. Until then, strain RPK1 can be obtained from the lab of S.W. and K.U. upon request.

## Abbreviations

BCA, bicinchoninic acid; BLAST, basic local alignment search tool; DAPI, 4’,6-diamidine-2-phenylindol; HPLC, high pressure liquid chromatography; ICP, inductively coupled plasma; KPN, potassium phosphate-sodium chloride; LB, lysogeny broth; Lcp, latex clearing protein; MALDI-ToF, matrix-assisted laser desorption ionisation time of flight; MS, mass spectrometry; MSM, mineral salts medium; NB, nutrient broth; ODTD, 12-oxo-4,8-dimethyl-trideca-4,8-diene-1-al; PHB, polyhydroxybutyrate; RoxA, rubber oxygenase A; SDS-PAGE, sodium dodecyl sulphate polyacrylamide gel electrophoresis; UVvis, ultra violet visible.

## Additional files

Additional file 1: Identity and similarity of biochemically characterized Lcp proteins and amino acid sequences alignment of biochemically characterized Lcp proteins. (DOCX 61 kb) Additional file 2: Detection of aldehyde products of Lcp-degraded polyisoprene by Fuchsin assay. (DOCX 1.91 kb) Additional file 3: Bipyridyl assay of Lcp_Rr_ in comparison to Lcp_K30_. (DOCX 128 kb) Additional file 4: UVvis spectra of Lcp_K30_ and Lcp_Rr_ in the presence of mercaptoethanol. (DOCX 356 kb)
