# Supplementary material for: Biochemical and spectroscopic characterization of purified Latex Clearing Protein (Lcp) from newly isolated rubber degrading Rhodococcus rhodochrous strain RPK1 reveals novel properties of Lcp
Source: BMC Microbiol. 2016 May 23;16:92. doi: 10.1186/s12866-016-0703-x (PMC4877957; doi:10.1186/s12866-016-0703-x)
Supplement: Additional file 1: — Identity and similarity of biochemically characterized Lcp proteins and amino acid sequences alignment of biochemically characterized Lcp proteins. (DOCX 61 kb) [file 12866_2016_703_MOESM1_ESM.docx]

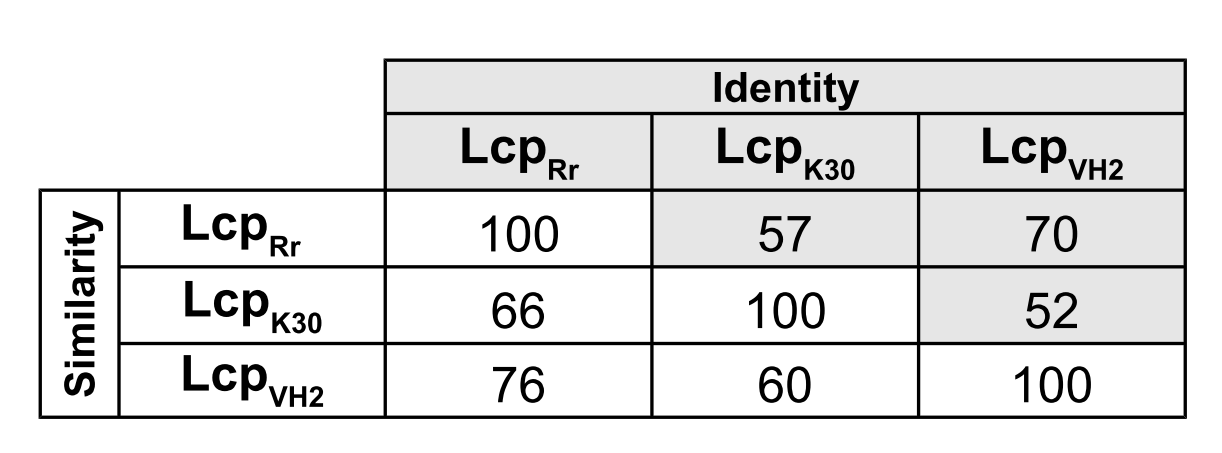


**additional file 1a**: Identity and similarity of biochemically characterise Lcp proteins.


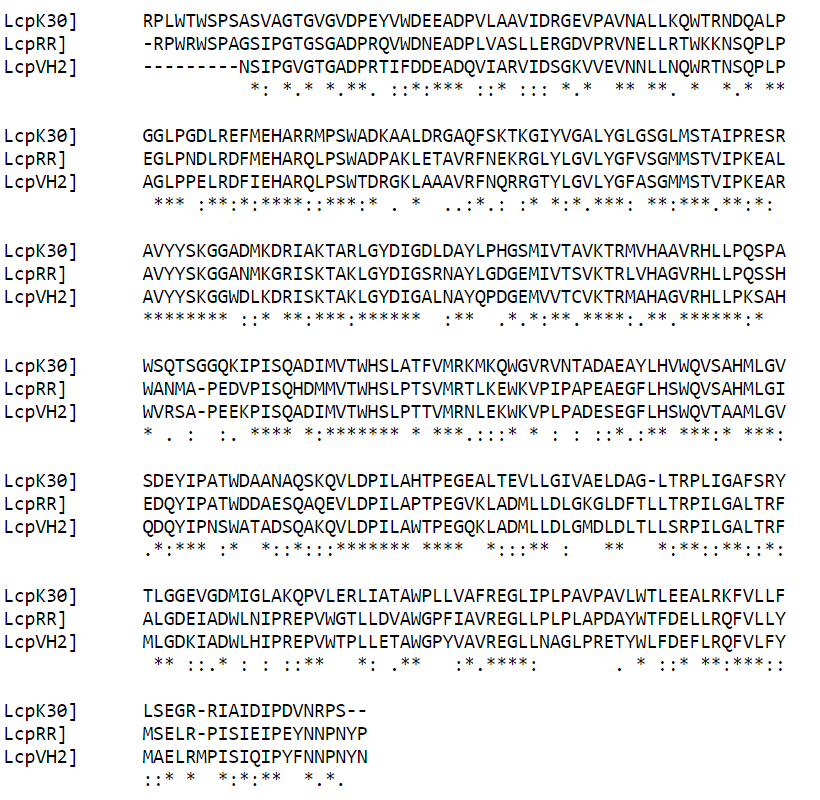


**additional file 1b:** Amino acid sequences alignment of biochemically characterised Lcp proteins (without signal peptide sequences). Asterics and colons indicate identical or similar residues, respectively.
