# Supplementary material for: Biochemical and spectroscopic characterization of purified Latex Clearing Protein (Lcp) from newly isolated rubber degrading Rhodococcus rhodochrous strain RPK1 reveals novel properties of Lcp
Source: BMC Microbiol. 2016 May 23;16:92. doi: 10.1186/s12866-016-0703-x (PMC4877957; doi:10.1186/s12866-016-0703-x)
Supplement: Additional file 2: — Detection of aldehyde products of Lcp-degraded polyisoprene by Fuchsin assay. (DOCX 1.91 kb) [file 12866_2016_703_MOESM2_ESM.docx]

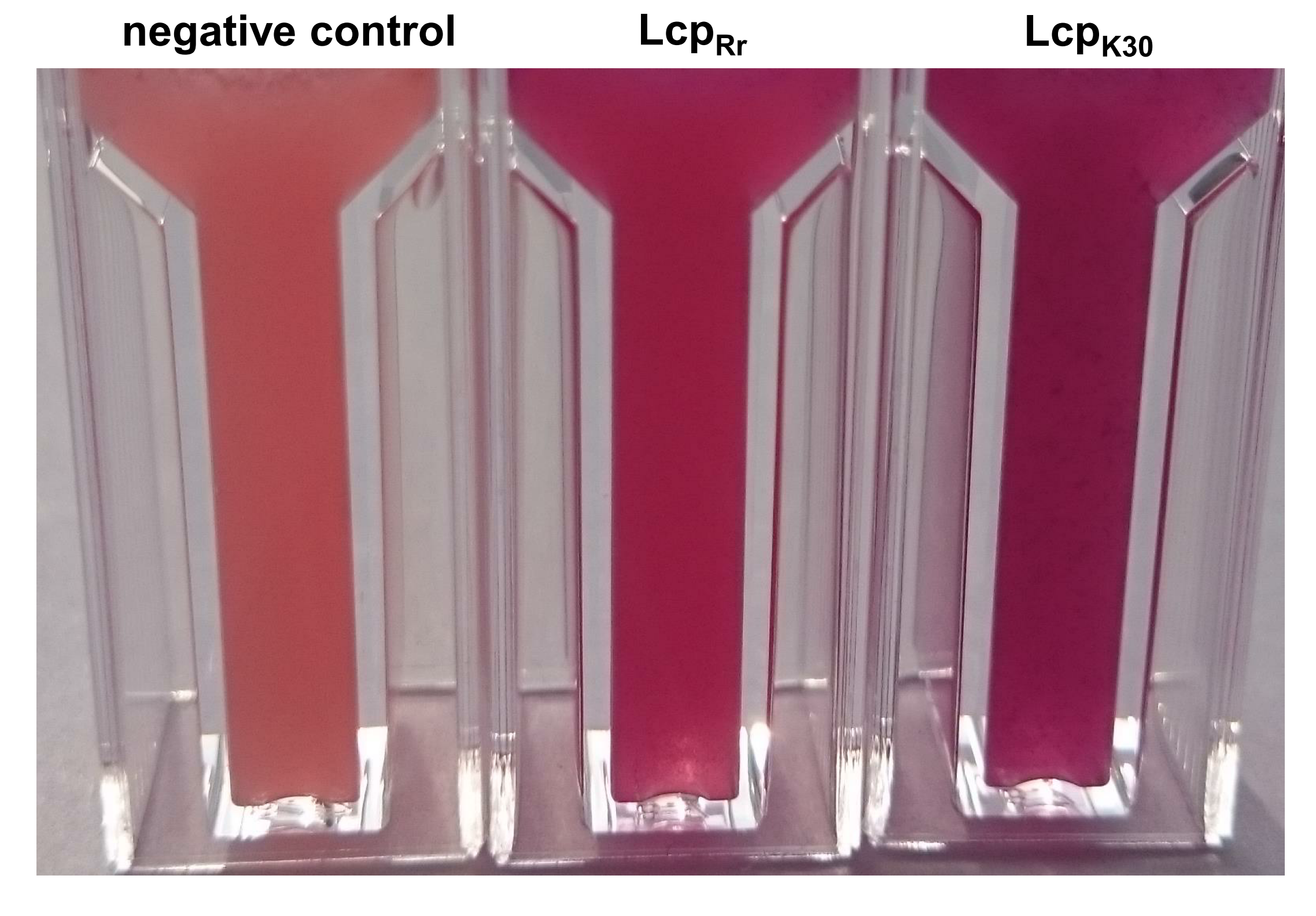


**Additional file 2:** Detection of aldehyde products of Lcp-degraded polyisoprene by Fuchsin assay. Polyisoprene latex in KP buffer, pH 8 was incubated with 4 µg/ml of purified Lcp_Rr_, 4 µg/mL of purified Lcp_K30_ or without enzyme (control) and incubated at 30°C for 1h. Development of pink color after addition of Fuchsin solution confirmed the formation of carbonyl groups in degradation products by Lcp_Rr_ and Lcp_K30_
