# Supplementary material for: Biochemical and spectroscopic characterization of purified Latex Clearing Protein (Lcp) from newly isolated rubber degrading Rhodococcus rhodochrous strain RPK1 reveals novel properties of Lcp
Source: BMC Microbiol. 2016 May 23;16:92. doi: 10.1186/s12866-016-0703-x (PMC4877957; doi:10.1186/s12866-016-0703-x)
Supplement: Additional file 3: — Bipyridyl assay of LcpRr in comparison to LcpK30. (DOCX 128 kb) [file 12866_2016_703_MOESM3_ESM.docx]

**
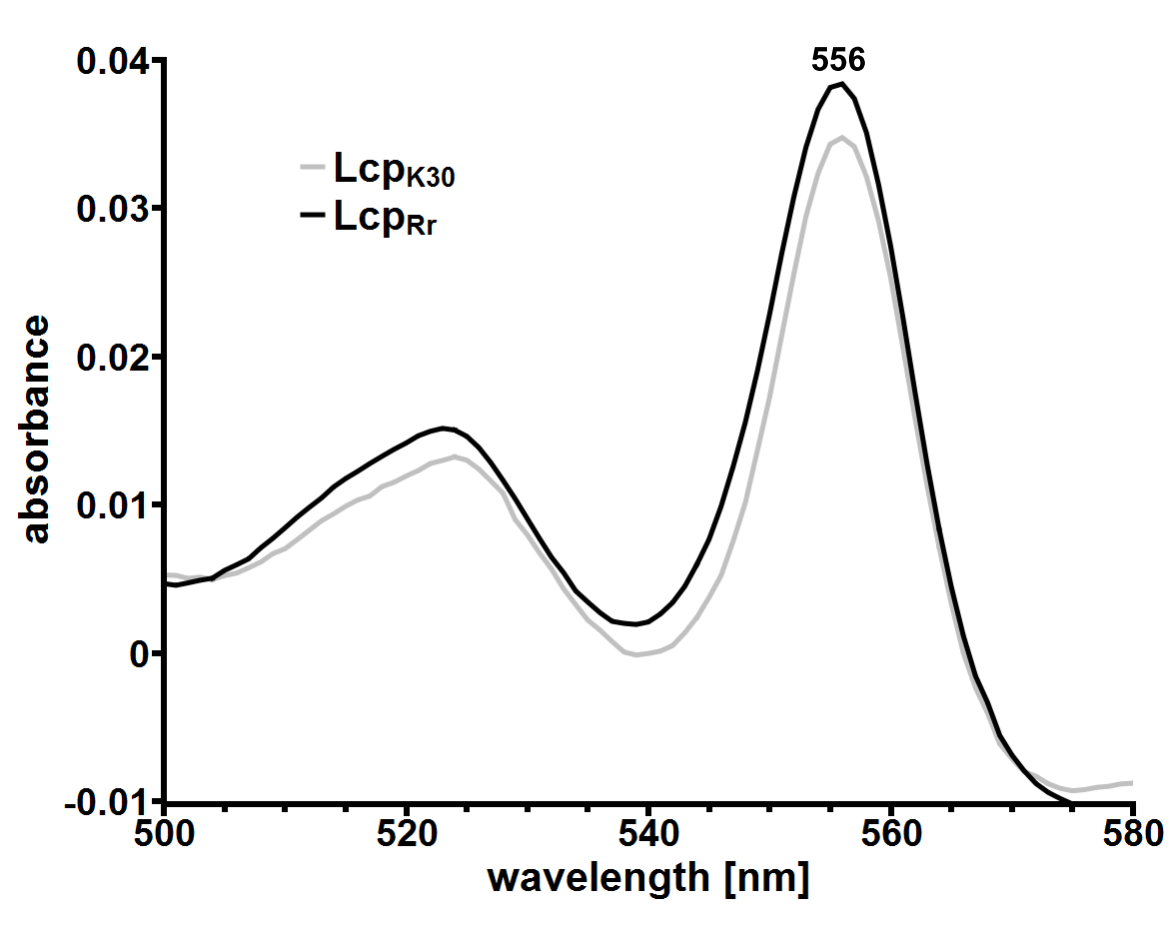
**

**Additional file 3:** Bipyridiyl assay of LcpRr (black line) in comparison to Lcp_K30_ (grey line). Note, the presence of an absorption maximum at 556 nm typical for a *b*-type cytochrome.
