# Supplementary material for: Biochemical and spectroscopic characterization of purified Latex Clearing Protein (Lcp) from newly isolated rubber degrading Rhodococcus rhodochrous strain RPK1 reveals novel properties of Lcp
Source: BMC Microbiol. 2016 May 23;16:92. doi: 10.1186/s12866-016-0703-x (PMC4877957; doi:10.1186/s12866-016-0703-x)
Supplement: Additional file 4: — UVvis spectra of LcpK30 and LcpRr in the presence of mercaptoethanol. (DOCX 356 kb) [file 12866_2016_703_MOESM4_ESM.docx]

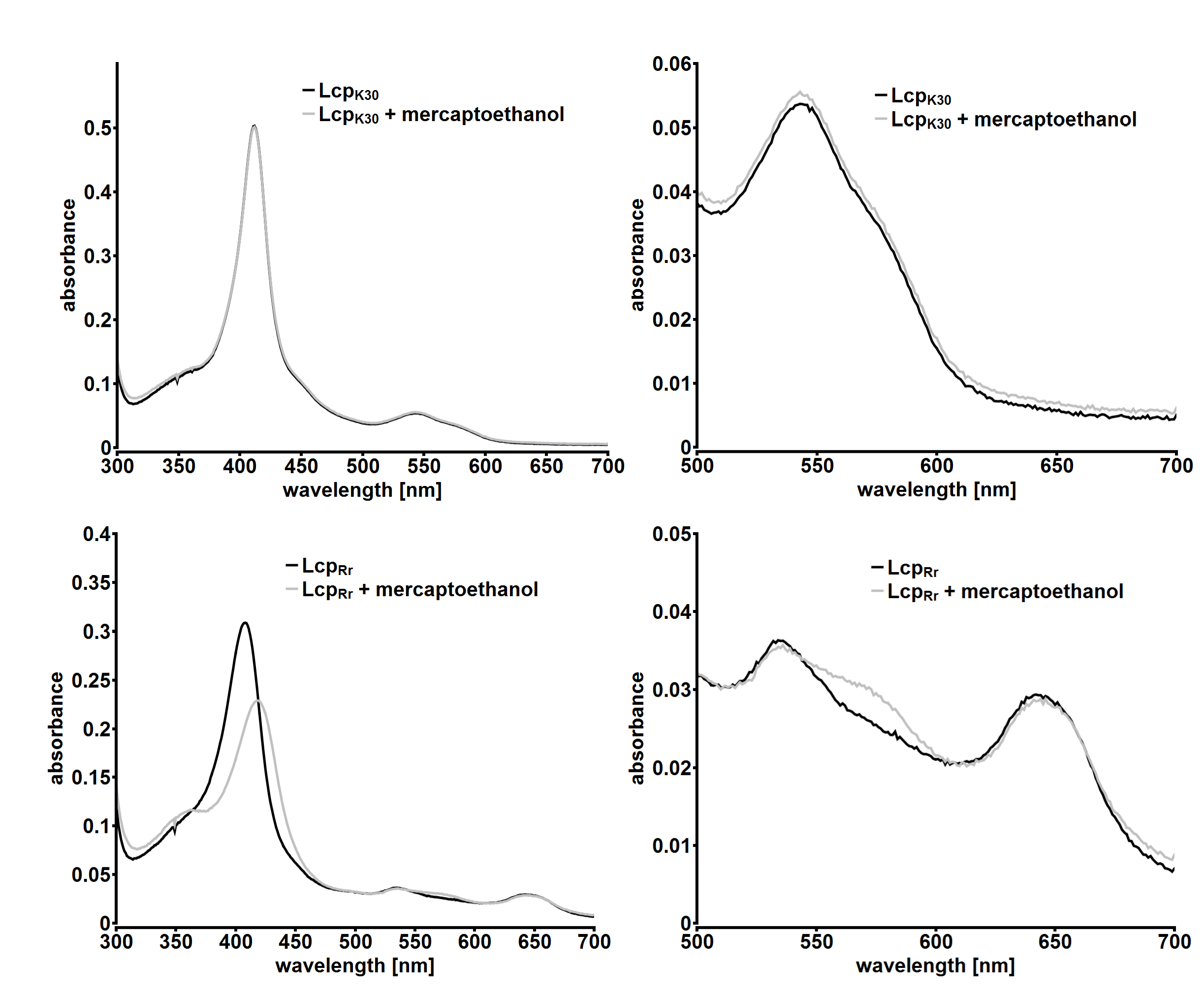


**Additional file 4:** UVvis spectra of Lcp_K30_ and Lcp_Rr_ in the presence of mercaptoethanol. Note, a change of the spectrum in the presence of mercaptoethanol only in case of Lcp_Rr_ but not in case of Lcp_K30._
